# Selective Nonthermal Melting in Phlogopite under Ultrafast Energy Deposition

**DOI:** 10.1021/acs.jpcc.5c06758

**Published:** 2025-11-05

**Authors:** Nikita Medvedev

**Affiliations:** † 86889 Institute of Physics, Czech Academy of Sciences, Na Slovance 1999/2, Praha 8, 182 00, Czech Republic; ‡ Institute of Plasma Physics, Czech Academy of Sciences, Za Slovankou 3, Praha 8, 182 00, Czech Republic

## Abstract

Phlogopite is a complex magnesium-rich mineral from the dark mica group, KMg_3_(AlSi_3_O_10_)­(OH)_2_. Its response to ultrafast excitation of its electronic system is studied using a hybrid model that combines tight-binding molecular dynamics with transport Monte Carlo and the Boltzmann equation. Simulations predict that at the deposited dose of ∼0.17 eV/atom (electronic temperature *T*_e_ ∼ 11,000 K), the first hydrogens start to migrate in the otherwise preserved lattice, transiently turning mica into a superionic state. At the dose of ∼0.4 eV/atom (*T*_e_ ∼ 13,000 K), Mg atoms start to diffuse like a liquid within stable sublattices of other elements, suggesting a superionic–superionic phase transition. At a dose of approximately 0.5 eV/atom (*T*_e_ ∼ 14,000 K), the entire atomic lattice destabilizes, disordering on a picosecond time scale. It is accompanied by the formation of defect energy levels inside the bandgap. At the dose of ∼0.9 eV/atom (*T*_e_ ∼ 16,000 K), the bandgap completely collapses, turning the material metallic (electronically conducting). At even higher doses, nonthermal acceleration of atoms heats the atomic system at ultrafast time scales; K and O elements are most affected, accelerating within a few tens of femtoseconds.

## Graphical abstract

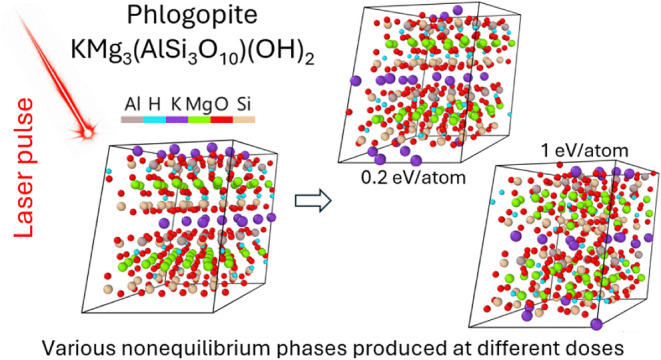

## 1 Introduction

Phlogopite (KMg_3_(AlSi_3_O_10_)­(OH)_2_), an example of dark mica, is a mineral with a monoclinic structure that forms flexible and elastic layers or flakes. The trioctahedral layers are connected by potassium sites; magnesium fills octahedral positions, whereas the tetrahedral sheets are occupied by a mix of silicon and aluminum atoms.^1^ It has low resistance to ionizing radiation (including gamma or beta rays, alpha particles, and heavy ions) under conventional low-dose-rate conditions.^2–4^ Phlogopite’s abundance in Earth’s crust instigated its various industrial and research applications, such as electrical and heat insulation, plastic and rubber reinforcement, use in construction materials, paints and coatings, as well as radiation-related applications.^5–8^

Phlogopite susceptibility to ionizing radiation is used in a geological dating method, registering recoil tracks created by the natural α-decay of U, Th, and their fission products.^4^ A swift ion (decay product) leaves a track of structurally modified material, a few nanometers in diameter and some microns in length.^4,9^ Such tracks form via a sequence of processes, starting with the excitation of electrons by the propagating ion and eventually converting into observable atomic disorder.^9,10^

Phlogopite’s layered structure is similar to the matrix of clay, which motivated research on its electron and γ radiation to study the candidate materials for radionuclide waste storage.^2,3^ Clay is considered a backfill material to prevent radionuclide migration. Understanding the governing mechanism of radiation damage in layered geological materials thus triggered research in phlogopite.^2^

Recently, exfoliation of the natural dark mica into ultrathin layers or flakes has found applications in dielectric layers for 2D-optoelectronics.^11^ The application of such devices implies exposure to electromagnetic radiation. Additionally, nanoelectronic production often involves laser patterning of materials to tailor their properties.^12^

The common effect in all these irradiation scenarios is that they are all initiated by the excitation of the electronic ensemble of the target.^10,13,14^ The electronic system, driven out of equilibrium, undergoes the electron cascades of secondary ionizations, thermalizing and exchanging the energy with the atomic system (the electron–phonon coupling).^13^ Atomic heating, overcoming the melting point, may lead to phase transitions, forming new material states.^15,16^ At the same time, high electronic excitation induces a modification of the interatomic potential, which may destabilize the lattice and lead to disruption of atomic bonds (also known as nonthermal melting).^17,18^

Quantitative understanding of the various effects leading to final material modifications is required for practical applications. It must necessarily include the nonequilibrium effects in both electronic and atomic systems, phase transitions, and chemical bond evolution, describing the physics and chemistry of the transient states outside the materials’ phase diagram. Ab initio methods, such as the density-functional method, are limited to small simulation boxes and usually do not include nonadiabatic electron–ion coupling.^19,20^ Classical molecular dynamics (MD) simulations, on the other hand, allow for a large system treatment but do not include electronic dynamics and nonthermal effects (changes in the interatomic potential due to electronic excitation).^21,22^

To study the phlogopite’s response to radiation and various damage induced by irradiation, the XTANT-3 code is used here.^23^ It combines a few approaches into a unified model with feedback: tight-binding molecular dynamics, transport Monte Carlo, and Boltzmann collision integral methods, delivering a state-of-the-art simulation method. This method enables to study electronic and atomic dynamics, modeling the intertwined effects of the thermal, nonthermal, and nonequilibrium kinetics in the irradiated phlogopite. Simultaneously, it is capable of modeling sufficiently large simulation boxes to capture the effects of a complex material.

## 2 Model

The hybrid code XTANT-3 includes the following approaches to trace the effects of irradiation: the transport Monte Carlo method to describe irradiation and kinetics of fast electrons and deep-shell holes; the Boltzmann equation to trace the slow electrons populating the valence band and the bottom of the conduction band; and the tight-binding molecular dynamics propagating the atomic trajectories on the evolving potential energy surface.^23^ All the numerical details of the simulation can be found in the XTANT-3 manual;^23^ here, the physical processes and models describing them are briefly outlined.

The electronic excitation induced by photoabsorption, the following nonequilibrium electron cascades, and the Auger decays of core holes are modeled with the event-by-event individual particle transport Monte Carlo method.^10^ The EPICS2025 database is used to extract the photoabsorption cross sections, the atomic ionization potentials, and Auger-decay times.^24^ The excited electrons perform elastic and inelastic collisions until they lose their energy below a predefined cutoff. Elastic scattering is described with the screened Rutherford scattering cross section with a modified Molier screening parameter.^25^ For inelastic scattering (impact ionizations and scattering on plasmons), the linear response theory is implemented with the single-pole approximation.^26^ The calculated electron inelastic mean free paths, as well as combined photoabsorption attenuation lengths, are listed in the Supporting Information.

Electrons with energies below the cutoff, populating the valence band and the bottom of the conduction band, are modeled with the Boltzmann collision integrals for the electron–electron and electron–phonon (electron–ion) scattering.^27^ The electron–electron interaction is described with the relaxation-time approximation; in this work, the electron relaxation time is set to instantaneous thermalization, which ensures that the electronic distribution function adheres to the Fermi–Dirac distribution. The effects of electronic nonequilibrium were studied in detail in ref 27. The nonadiabatic electron–ion energy exchange is calculated with the dynamical coupling method.^28^

The valence- and conduction-band energy levels (band structure) evolve with the transferable tight-binding method.^29–31^ The sp^3^d^5^-based PTBP density-functional tight-binding parametrization is used here, covering the pairwise interaction of all the elements involved.^32^ The diagonalization of the electronic Hamiltonian produces the electronic energy levels (molecular orbitals) and the transient interatomic forces, which are dependent on the relative positions of all the atoms in the simulation box.^29^ The electronic distribution function (traced with the Boltzmann equation) directly affects the interatomic potential, enabling the description of the nonthermal melting and the effects of bond breaking.^31,33^

The atomic motion is traced with the molecular dynamics simulation, applying Martyna–Tuckerman’s fourth-order algorithm with a time step of 0.2 fs.^34^ The simulation box contains 396 atoms (3 × 3 × 2 unit cells, 1.81 × 1.46 × 2.07 nm^3^—sufficiently large to eliminate finite-size effects),^28^ with the unit cell taken from ref 35, relaxed via the steepest descent algorithm, producing an equilibrium density of 2.73 g/cm^3^. Then, the atomic velocities are initialized with the Maxwellian distribution at room temperature, allowing the material to thermalize before the arrival of the radiation pulse of 92 eV photon energy and 10 fs (fwhm) duration.

Standard methods to describe nonthermal effects are based on density functional theory (DFT). Previous comparisons between XTANT-3 simulations and DFT showed reasonably good agreement within the Born–Oppenheimer approximation (a necessary approximation in DFT models).^36^ Time-dependent DFT also validated nonadiabatic effects predicted with XTANT-3.^37^

The illustrations of the atomic snapshots are prepared with the help of OVITO software.^38^

## 3 Results

This section starts by evaluating the phlogopite electronic density of states (DOS), since the electronic properties affect the atomic potential and will further help to analyze the atomic behavior. The total and partial (projected) DOS in phlogopite are shown in Figure 1. They are evaluated on the 7 × 7 × 7 k-point Monkhorst–Pack grid in the entire supercell (396 atoms).^39^ The valence band is mainly formed by the *p*-states of oxygen atoms, whereas the bottom of the conduction band has significant contributions from K, Mg, Si, and Al atoms (the H contribution is minor).

**Figure 1:**
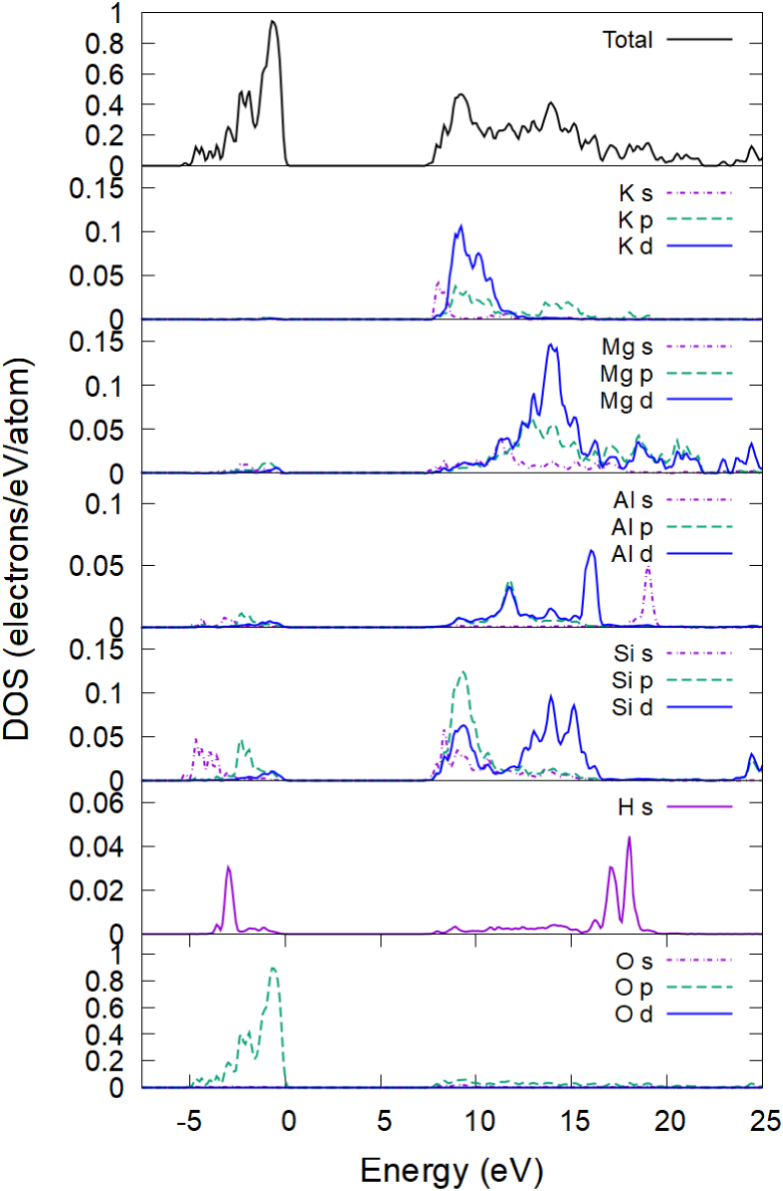
Total and partial electronic density of states in phlogopite is counted from the Fermi level.

The calculated DOS qualitatively agrees with the previously reported DFT calculations.^40^ The calculated band gap in the ideal crystal structure is 7.5 eV and shrinks to ∼6.5 eV at room temperature, which also agrees reasonably well with various studies reporting the gap values between 4.8 and 6.9 eV.^11,41^ This result validates applicability of the used PTBP tight-binding parametrization to phlogopite.^32^

The irradiation of phlogopite was performed using an ultrashort laser pulse of 92 eV photon energy, 10 fs fwhm duration, and various deposited doses (or energy densities—the terms are used interchangeably in this work) to identify the damage mechanisms and thresholds. The absorbed dose of 0.17 ± 0.02 eV/atom (corresponding to a peak electronic temperature of *T*_e_ ∼ 11,000 K, Figure 2) induces first defects: hydrogen migration, see Figure 2 showing atomic displacements of various species. The mean displacement of hydrogen grows continuously, whereas that of other elements saturates, indicating a stable lattice. Hydrogen diffuses, crossing the Mg layer, as shown in Figure 3. During this time, the electronic and atomic temperatures are still out of equilibrium. At this deposited dose, their equilibration requires tens of picoseconds, defined by the coupling parameter (see the bottom panel of Figure 2), which is relatively small.

**Figure 2:**
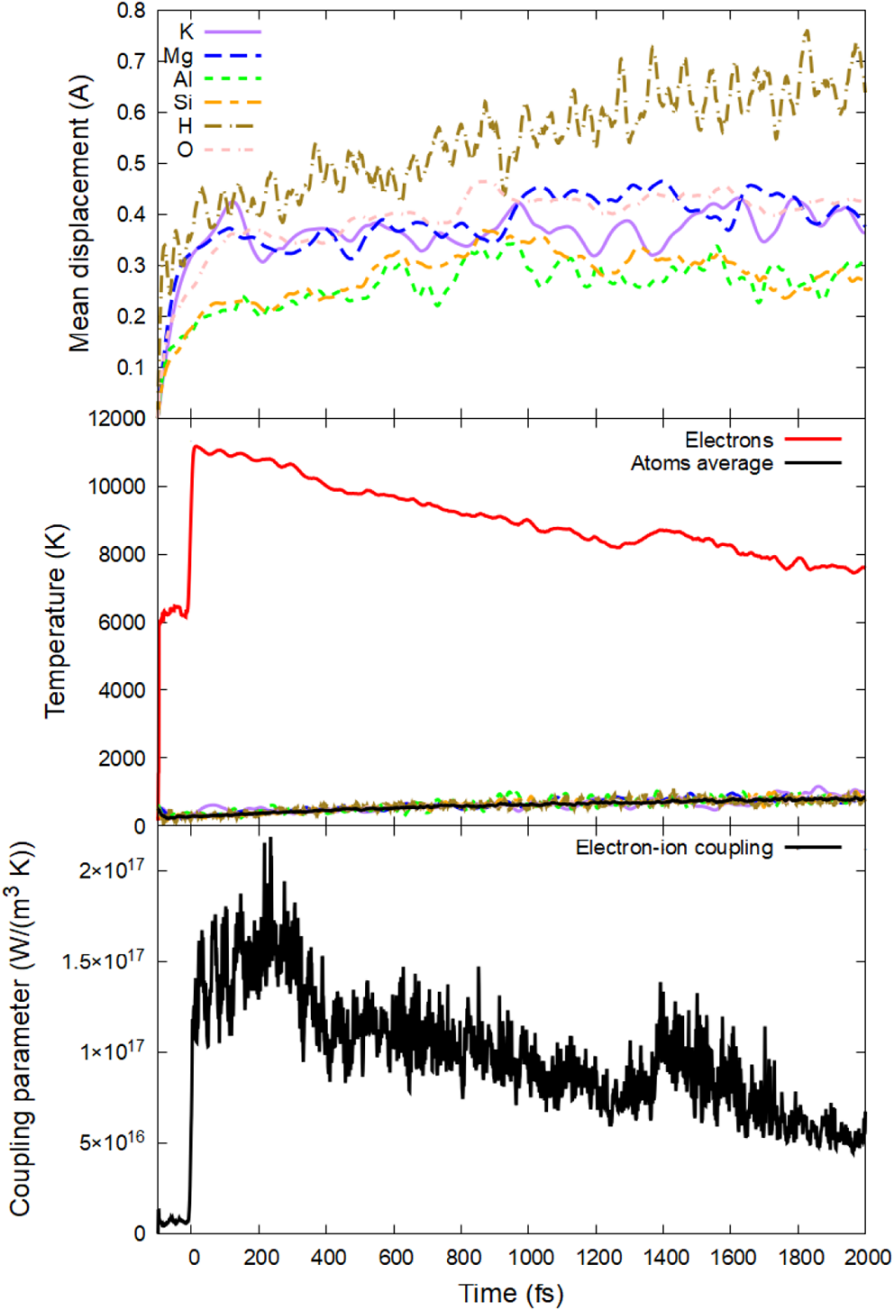
(Top panel) Mean displacements of different elements; (middle panel) electronic and atomic temperature (total and element-resolved); (bottom panel) electron–phonon coupling parameter in phlogopite irradiated with 0.17 eV/atom, 92 eV photon energy, and 10 fs fwhm duration.

**Figure 3:**
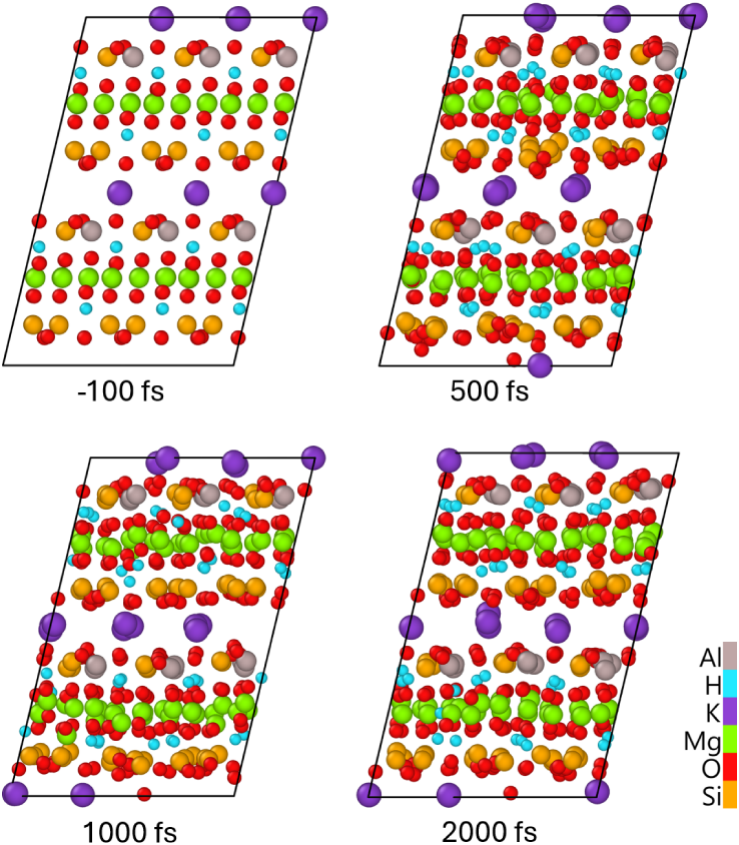
Atomic snapshots of phlogopite irradiated with 0.17 eV/atom, 92 eV photon energy, and 10 fs fwhm duration, simulated within the nonadiabatic approximation (electron–phonon coupling included).

It is interesting to note that in a Born–Oppenheimer simulation (excluding electron–phonon coupling), the first damage occurs at a lower dose of 0.15 ± 0.01 eV/atom (∼0.4% of valence electrons excited to the conduction band) via Mg and Si atom displacements into different planes, see Figure 4. This difference from the nonadiabatic simulation (cf. Figure 3) appears to be due to the electronic temperature being kept higher in the BO simulation than in the non-BO one (Figure 4 vs Figure 2): without the electron–phonon coupling, the electronic temperature stays constant.

**Figure 4:**
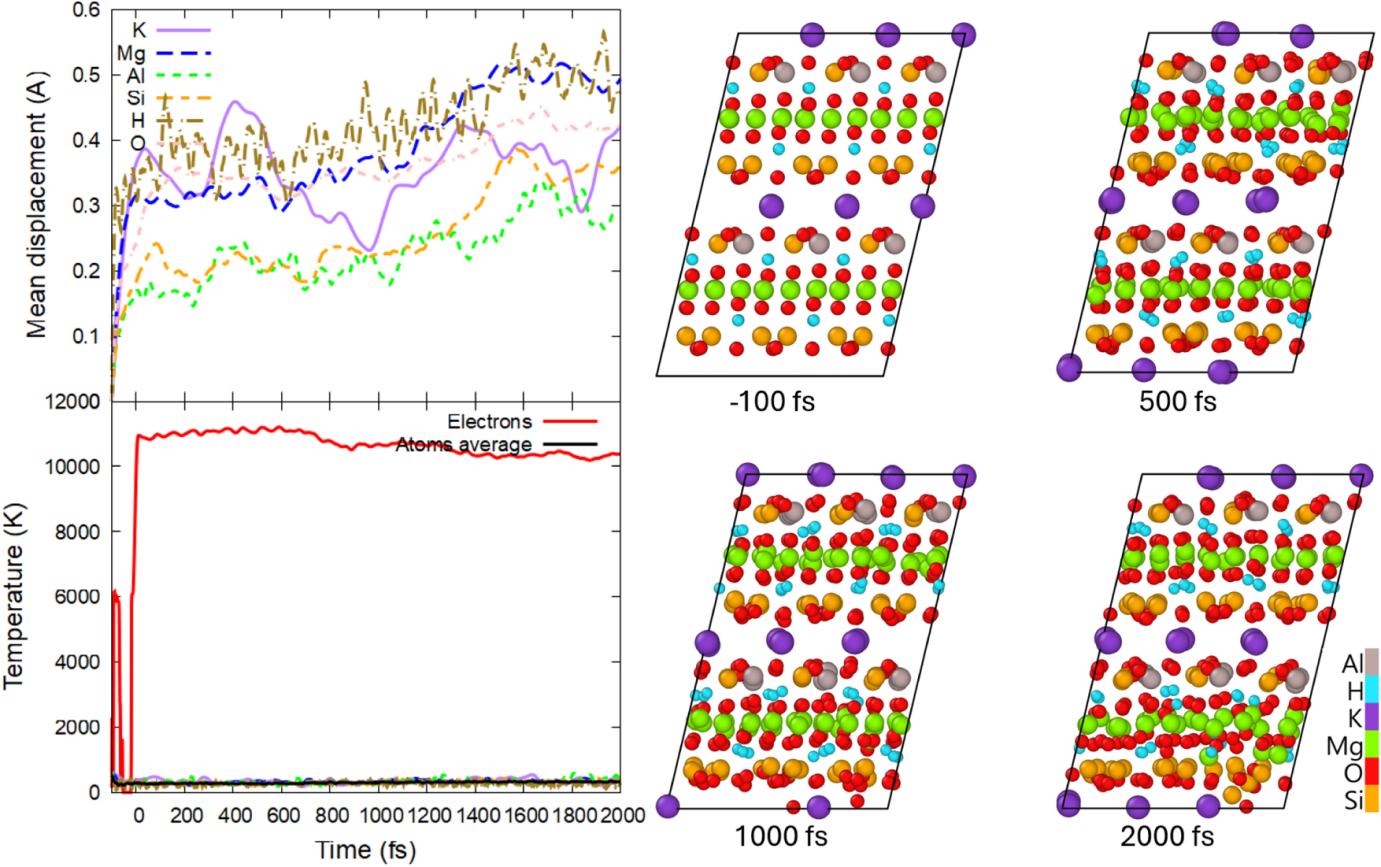
(Top left panel) Mean displacements of different elements; (bottom left panel) electronic and atomic temperature (total and element-resolved); (right panels) atomic snapshots of phlogopite irradiated with 0.15 eV/atom, 92 eV photon energy, and 10 fs fwhm duration, simulated with the BO approximation (excluding electron–phonon coupling).

The nonadiabatic (electron–phonon coupling) and nonthermal (changes of the interatomic potential due to electronic excitation) effects are competing in phlogopite. The nonthermal melting is caused by significant electronic excitation from the valence band to the conduction band of the material. A large number of electrons in the conduction band weakens the interatomic bonds. Electronic cooling due to electron–phonon coupling includes their relaxation back from the conduction band to the valence band. Such relaxation reduces the number of electrons in the conduction band, thereby restoring the original interatomic potential and precluding nonthermal damage in the Mg and Si subsystems, increasing the damage threshold. A similar effect of increasing the damage dose via electron–phonon coupling was observed in diamond.^42^

In the non-BO simulation, Mg atoms start to displace at a dose of 0.4 ± 0.05 eV/atom (*T*_e_ ∼ 13,000 K, see Figure 5). At such doses, diffusion of the Mg atoms occurs within stable sublattices of other elements. In this state in phlogopite, Al, K, O, and Si form a solid lattice, whereas H and Mg are liquid-like. It suggests that around this deposited dose, a superionic–superionic phase transition takes place—from a state with liquid-like hydrogen to a two-liquid-subsystems state.

**Figure 5:**
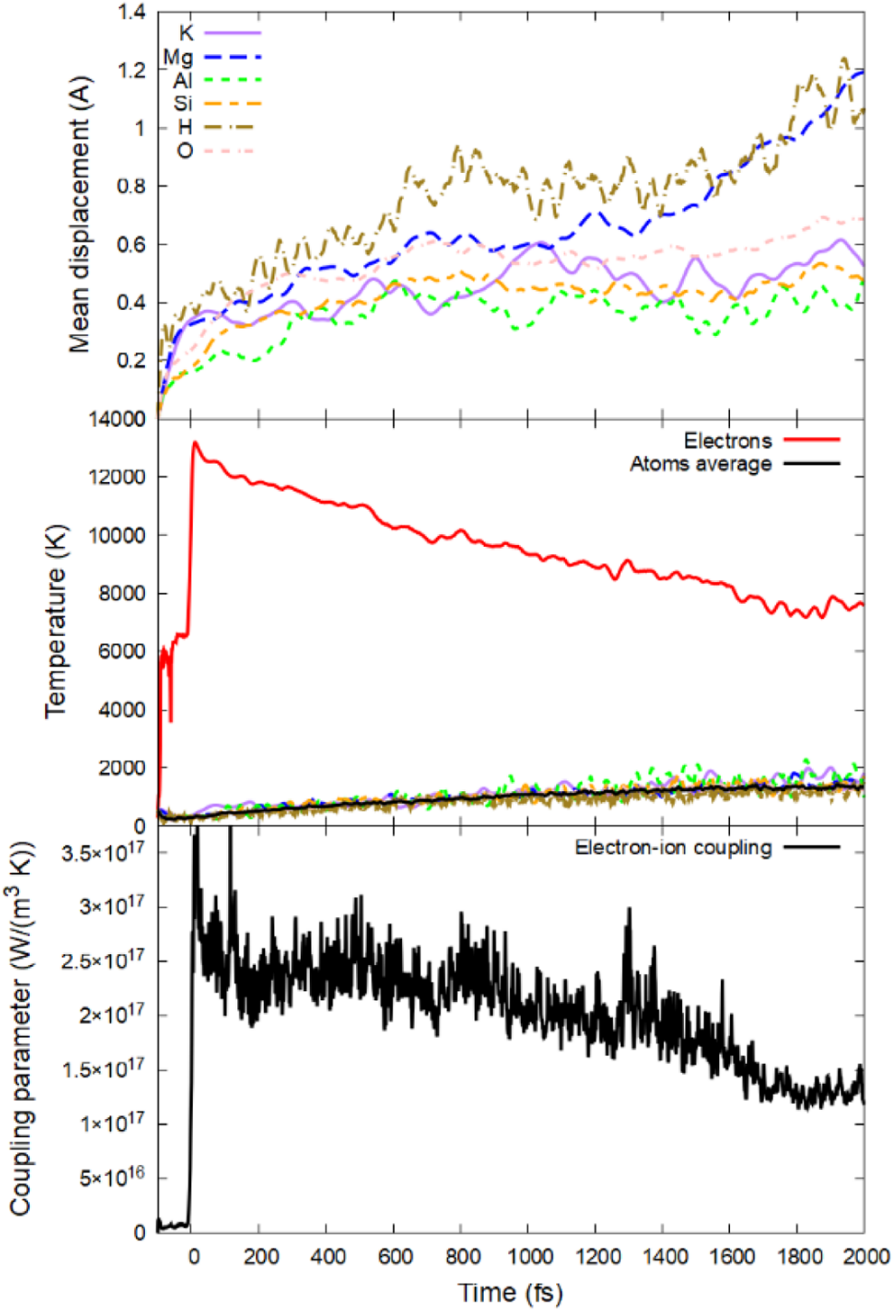
(Top panel) Mean displacements of different elements; (middle panel) electronic and atomic temperature (total and element resolved); (bottom panel) electron–phonon coupling parameter in phlogopite irradiated with 0.4 eV/atom, 92 eV photon energy, and 10 fs fwhm duration.

With an increase of the dose to 0.5 ± 0.05 eV/atom (*T*_e_ ∼ 14,000 K), the entire atomic lattice destabilizes, disordering at a few-ps time scale, see Figure 6. Nonetheless, the complete melting of the system occurs with different sublattices disordering at different rates, with H and Mg subsystems disordering the fastest, whereas Al and K are the slowest. The phlogopite disorder is accompanied by the formation of defect energy levels inside the band gap, see Figure 7.

**Figure 6:**
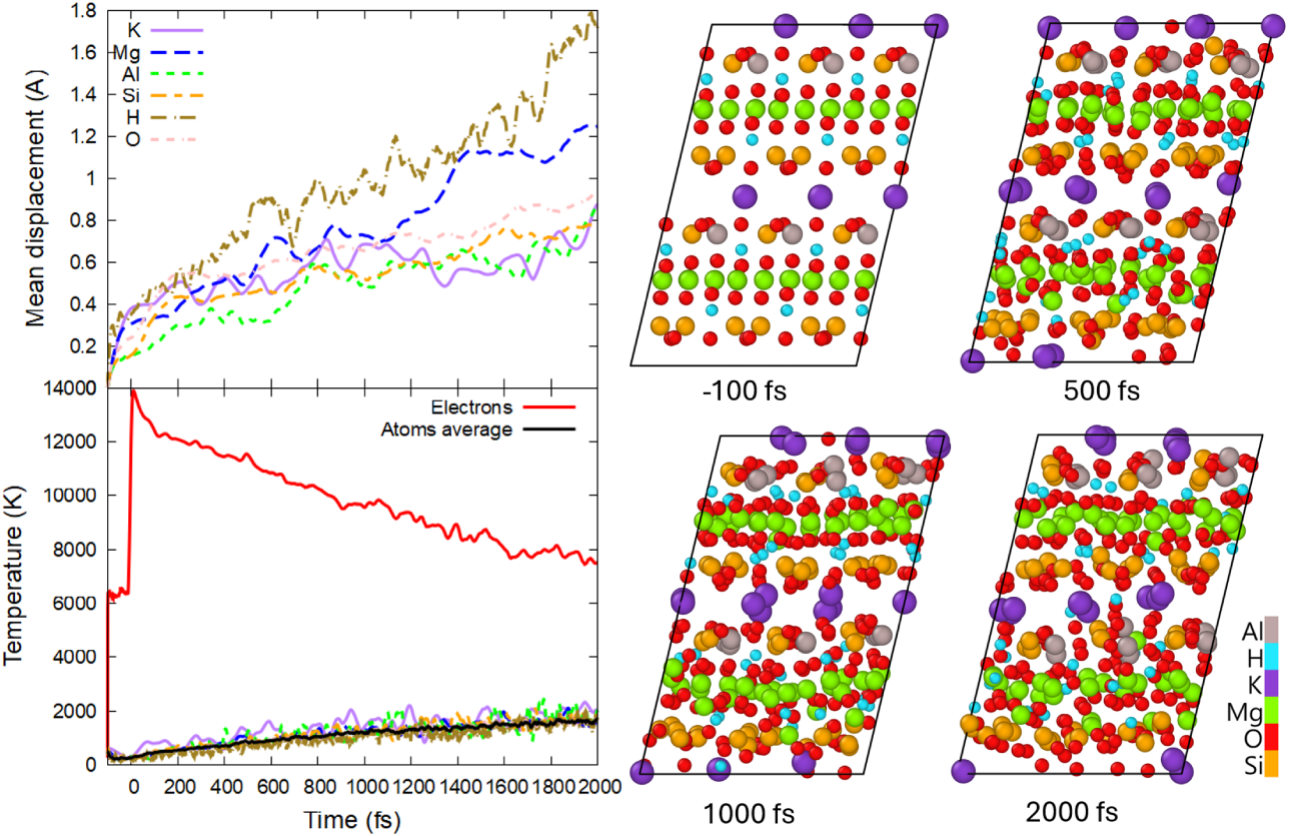
(Top left panel) Mean displacements of different elements; (bottom left panel) electronic and atomic temperature (total and element-resolved); (right panels) atomic snapshots in phlogopite irradiated with 0.5 eV/atom, 92 eV photon energy, and 10 fs fwhm duration.

**Figure 7:**
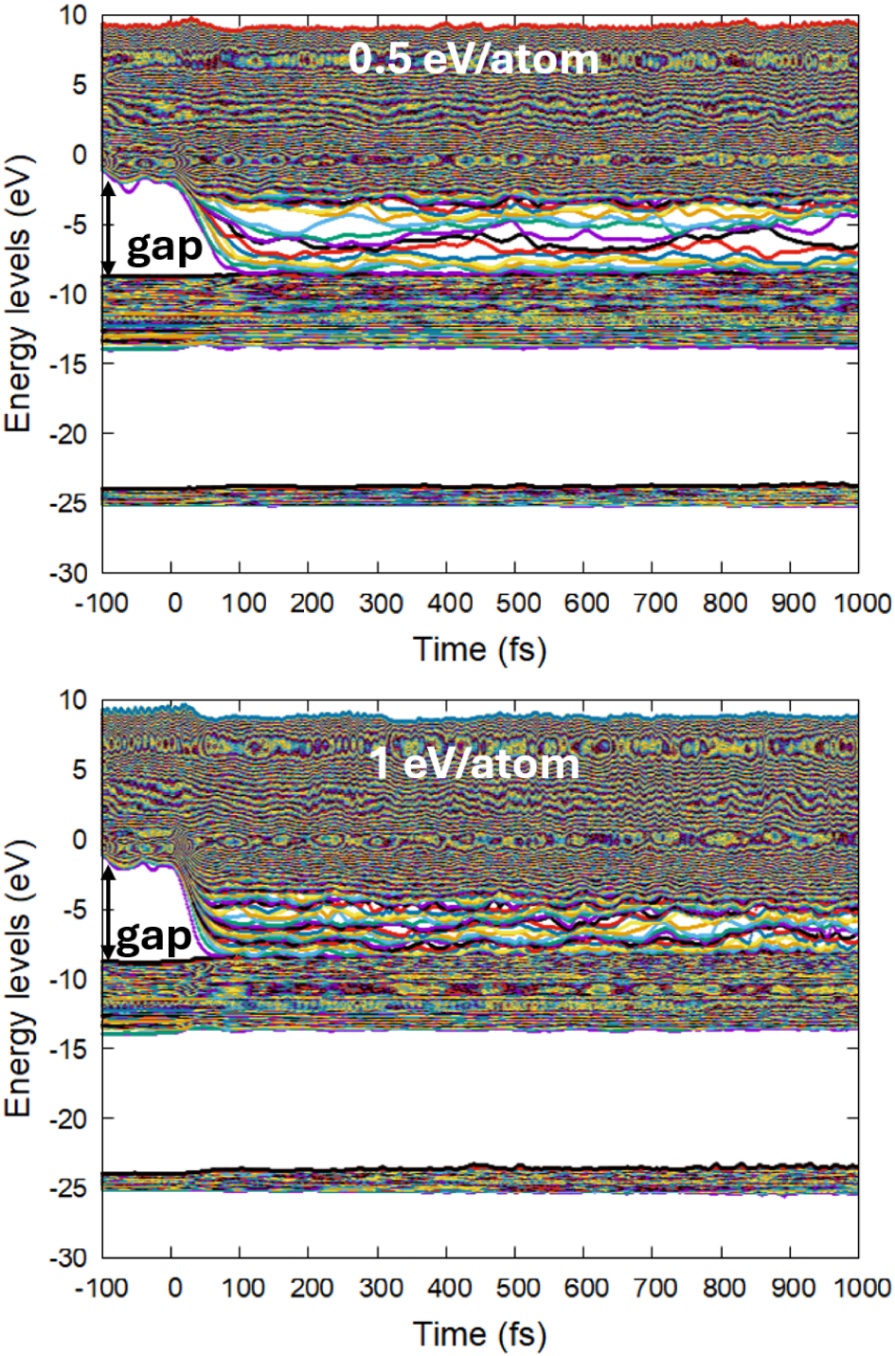
Electronic energy levels (molecular orbitals) in phlogopite irradiated with a pulse of 92 eV photons, 10 fs fwhm duration, and an absorbed dose of 0.5 eV/atom (top panel) and 1 eV/atom (bottom panel). The initial band gap is indicated with the arrows.

Above the dose of ∼0.9 ± 0.1 eV/atom (*T*_e_ ∼ 16,000 K), the bandgap completely collapses (bottom panel in Figure 7), turning the material metallic (electronically conducting). Thus, a different liquid nonequilibrium phase may be produced in phlogopite compared to the lower doses discussed above. It may be concluded that the transient states in phlogopite may be superionic, semimetallic, or metallic, depending on the deposited dose; completely disordered phlogopite is metallic. Upon sufficiently fast cooling, the state may be quenched to an amorphous phase.

At even higher doses, nonthermal acceleration heats the atomic system at subpicosecond time scales, see an example of a 2 eV/atom dose in Figure 8 (peak electronic temperature ∼ 20,000 K). The fastest element to accelerate is oxygen, followed closely by potassium and then other elements. These elements acquire high kinetic temperatures within a few tens of femtoseconds (the temperature defined in the MD simulation via average kinetic energy of atoms^43^). The increase in velocities leads to the rise in atomic displacements as the system loses its stability and structure.

**Figure 8:**
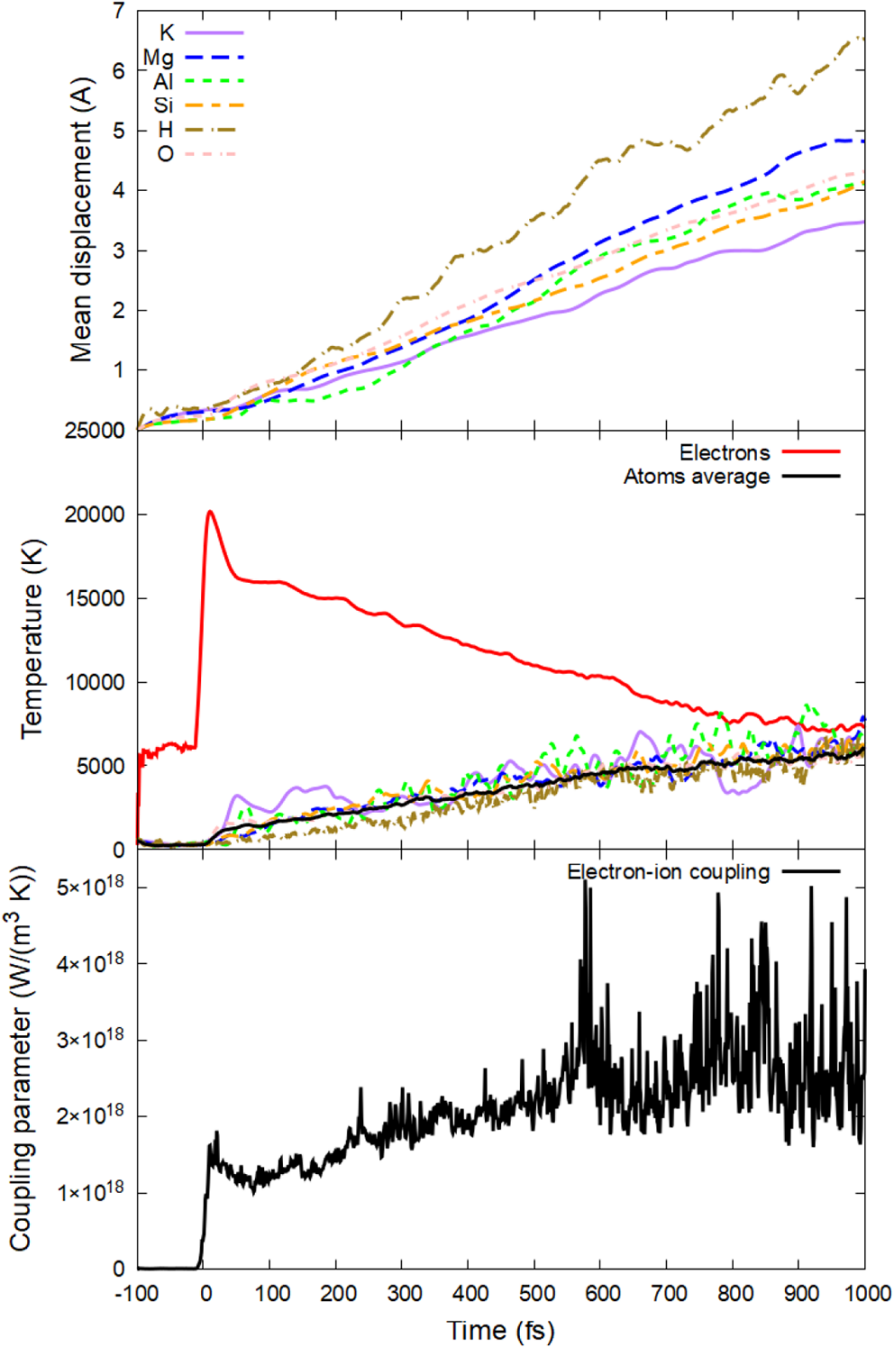
(Top panel) Mean displacements of different elements; (middle panel) electronic and atomic temperature (total and element resolved); (bottom panel) electron–phonon coupling parameter in phlogopite irradiated with 2 eV/atom, 92 eV photon energy, and 10 fs fwhm duration.

This selective atomic acceleration may be qualitatively explained by the electronic DOS structure (recall Figure 1): the electronic temperature increase smears out the Fermi–Dirac distribution function, removing electrons from the top of the valence band and promoting them to the bottom of the conduction band. The top of the valence band is predominantly formed by the oxygen *p*-states. As electrons are removed from there, the interatomic potential acting on oxygen atoms weakens and eventually turns more repulsive.^44,45^ The bottom of the conduction band has large contributions from K (and Al, Si) states—as electrons are promoted into these states, the corresponding elements experience the modified interatomic potential and accelerate too.

As discussed in detail in ref 46, nonthermal atomic acceleration leads to an increase in the electron–phonon coupling parameter (which is proportional to the atomic temperature). The electron–phonon coupling is stronger at this irradiation dose than at lower ones (bottom panels in Figure 8 vs Figures 2 and 5). This self-amplifying process of nonthermal atomic acceleration, reinforcing atomic heating via electron–phonon coupling, leads to a complete atomic disorder on the scale of a few hundred femtoseconds.

## 4 Discussion

The experiments on β- and γ-irradiation of phlogopite reported damage occurring at a dose on the order of 1000 kGy, at which significant hydrogen migration takes place, leading to material swelling.^2,3^ Considering the density of phlogopite and its average atomic mass, this dose converts to ∼0.2 eV/atom, which is remarkably close to the damage threshold of 0.17 eV/atom calculated in the present study. In the calculations, this damage is also associated with hydrogen bond breaking, detachment, and diffusion. These results validate the simulation.

In experiments on irradiation of phlogopite with swift heavy ions in refs 47,48 , the ion tracks were visualized with the help of chemical etching. The etched tracks had two distinct shapes: at lower ion energy loss, they were laterally triangular and discontinuous in depth, whereas with an increase in the energy loss of the projectile, the etch pits turned into a continuous hexagonal shape.^47,48^ This indicates that two different damage mechanisms occur at different deposited doses. It was suggested that the hexagonal shape is associated with the damage in the SiO_4_ tetrahedron in phlogopite, whereas the triangular shape is formed by the hydroxyl group. Although these results are not directly comparable to the present simulations, qualitatively, they support the conclusions drawn that damage in the hydrogen-containing sublattice forms at lower doses than in the silicon-containing one. As a swift heavy ion excites the material in its path highly inhomogeneously, various doses (energy densities) occur at various radii around the ion path. Thus, all the effects reported here for the laser irradiation are expected to occur in ion tracks too.

Finally, after the damage thresholds for various processes in terms of the absorbed dose are calculated, the corresponding thresholds in terms of the incident photon fluence may be estimated. For this conversion, it is assumed that the incident pulse is normal to the surface of the sample, there is no reflection (typical for XUV or X-rays), there is no significant energy transport within the sample before the damage occurs, and there is no emission of particles and energy from the surface. Under such assumptions, the damage threshold fluence is evaluated as *F = D*λ*n*_at_, where *D* is the threshold dose, λ is the photon attenuation length at given photon energy (see Supporting Information), and *n*_at_ is the atomic concentration in the sample. The damage threshold fluences in phlogopite for hydrogen migration, superionic H and Mg subsystems, and complete disorder are shown in Figure 9. These results may help to guide future experiments on photopite irradiation with X-ray pulses.

**Figure 9:**
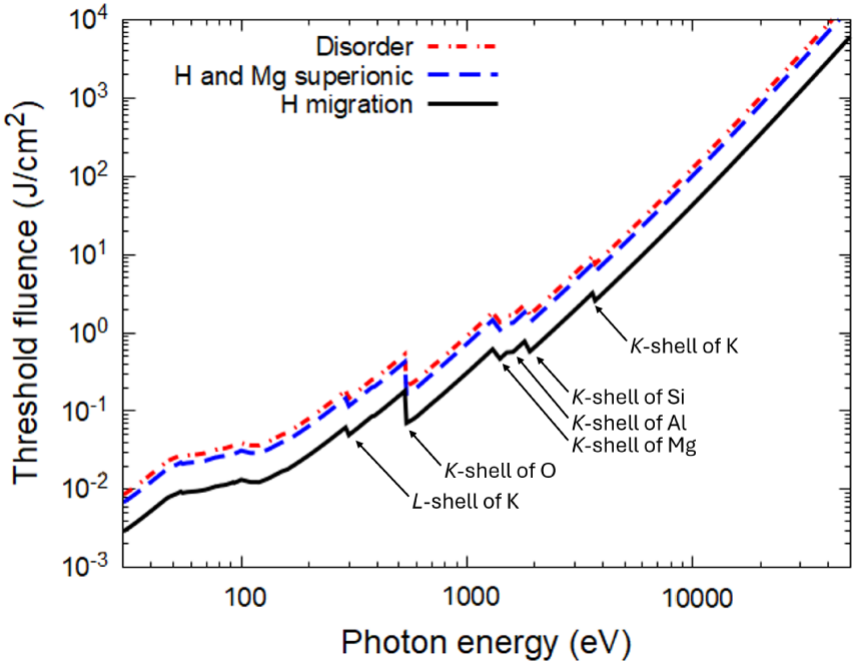
Damage threshold fluences in phlogopite for H migration, superionic H and Mg subsystem creation, and complete disorder. Contribution to photoabsorption from different shells of various elements, producing sudden jumps in the curves (marked with arrows).

The model used here relies on periodic boundary conditions, neglecting the energy and particle transport inside the material, as well as possible emission from the surface. These effects may need to be considered for particular experimental conditions: e.g., grazing incidence irradiation or a photon energy in the XUV range, which create an extremely short attenuation length and high spatial gradients inducing strong transport effects.^49^ Energy sinks from the irradiated regions increase the damage threshold with respect to that reported here. To some degree, the transport effects may be mimicked via using thermostats in the simulation, which is beyond the scope of the present work.^50^

## 5 Conclusions

Phlogopite (KMg_3_(AlSi_3_O_10_)­(OH)_2_) under electronic excitation is modeled with a combined code, XTANT-3. Combining tight-binding molecular dynamics with transport Monte Carlo and the Boltzmann equation enables the study of the nonequilibrium, nonthermal, and nonadiabatic effects in realistically complex materials.

Simulations predict that at the threshold dose of 0.17 eV/atom (electronic temperature *T*_e_ ∼ 11,000 K), the first hydrogens detach and migrate. This estimated dose is in good agreement with the experimentally measured one, 1000 kGy = 0.2 eV/atom. Curiously, the nonthermal and nonadiabatic effects in phlogopite are working in opposite directions: electron–phonon coupling, cooling the electronic system, precluding the nonthermal damage in the atomic system, and increasing the damage threshold with respect to the Born–Oppenheimer simulation.

Increasing the deposited dose to 0.4 eV/atom (*T*_e_ ∼ 13,000 K), Mg atoms start to diffuse like a liquid, whereas other sublattices remain stable, thereby forming a different superionic state. Above the dose of 0.5 eV/atom (*T*_e_ ∼ 14,000 K), the entire atomic system disorders. At the doses of 0.9 eV/atom (*T*_e_ ∼ 16,000 K), the electronic bandgap completely collapses, forming a liquid metallic state. At even higher doses (∼2 eV/atom), nonthermal heating of the atomic system occurs at femtosecond time scales. This nonthermal effect is most pronounced in the K and O elements, selectively accelerating them.

The simulation results suggest that a rich variety of transient states exist in irradiated phlogopite. Tuning the irradiation dose may enable production, tailoring, and studying of such states.

## Supplementary Material



## Data Availability

The code XTANT-3 is publicly available from ref 23.

## Notes

Figure S1. Photon attenuation length (left panel) and electronic inelastic mean free path (right panel) in phlogopite (PDF)
