# Supplementary material for: Selective Nonthermal Melting in Phlogopite under Ultrafast Energy Deposition
Source: J Phys Chem C Nanomater Interfaces. 2025 Nov 5;129(46):20840–7. doi: 10.1021/acs.jpcc.5c06758 (PMC12641468; doi:10.1021/acs.jpcc.5c06758)
Supplement: Supplementary file 1 [file jp5c06758_si_001.pdf]

Supporting Information
for
"Selective nonthermal melting in phlogopite under ultrafast energy deposition"

Nikita Medvedev[1,2,*]

1) Institute of Physics, Czech Academy of Sciences, Na Slovance 1999/2, 182 00 Prague 8, Czech Republic
2) Institute of Plasma Physics, Czech Academy of Sciences, Za Slovankou 3, 182 00 Prague 8, Czech Republic

The total and partial photon attenuation lengths in the phlogopite, constructed from the EPICS2025 database [1] (for core shells) accounting for the stoichiometry of the material, and CDF-based cross section (for the valence band) are shown in Figure S1 (left panel) [2]. The electronic inelastic mean free paths in the same material, calculated with the linear response theory using the single-pole approximation, accounting for plasmon scattering [2], are shown in Figure S1 (right panel).

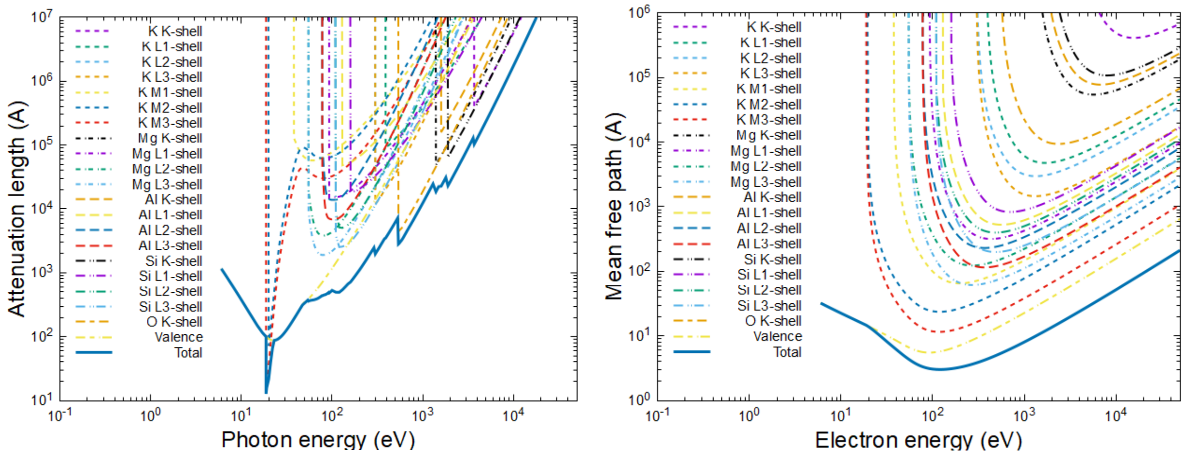

Figure S1. Photon attenuation length (left panel) and electronic inelastic mean free path (right panel) in phlogopite. Total values are shown in solid lines. Partial ones, for each atomic shell of each element, are differently styled dashed.

References

[1] "EPICS2025" https://nuclear.llnl.gov/EPICS/index.html (accessed 2025-09-25).

[2] N. Medvedev, F. Akhmetov, R.A. Rymzhanov, R. Voronkov, and A.E. Volkov, "Modeling time-resolved kinetics in solids induced by extreme electronic excitation," Adv. Theory Simulations **5**(8), 2200091 (2022).

[*] Corresponding author: email: nikita.medvedev@fzu.cz, ORCID: 0000-0003-0491-1090
